# Aggregate-level lead exposure, gun violence, homicide, and rape

**DOI:** 10.1371/journal.pone.0187953

**Published:** 2017-11-27

**Authors:** Brian B. Boutwell, Erik J. Nelson, Zhengmin Qian, Michael G. Vaughn, John P. Wright, Kevin M. Beaver, J. C. Barnes, Melissa Petkovsek, Roger Lewis, Mario Schootman, Richard Rosenfeld

**Affiliations:** 1 School of Social Work, Saint Louis University, St. Louis, Missouri, United States of America; 2 Department of Epidemiology and Biostatistics, Saint Louis University, St. Louis, Missouri, United States of America; 3 Department of Epidemiology and Biostatistics, Indiana University – Bloomington, Bloomington, Indiana, United States of America; 4 School of Criminal Justice, University of Cincinnati, Cincinnati, Ohio, United States of America; 5 Center for Social and Humanities Research, King Abdulaziz University, Jeddah, Saudi Arabia; 6 College of Criminology and Criminal Justice, Florida State University, Tallahassee, Florida, United States of America; 7 Department of Criminal Justice, University of Central Missouri, Warrensburg, Missouri, United States of America; 8 Department of Environmental and Occupational Health, Saint Louis University, St. Louis, Missouri, United States of America; 9 Department of Criminology and Criminal Justice, University of Missouri-St. Louis, St. Louis, Missouri, United States of America; Stony Brook University, Graduate Program in Public Health, UNITED STATES

## Abstract

**Context:**

An increasing body of research has linked the geographic distribution of lead with various indicators of criminal and antisocial behavior.

**Objective:**

The current study, using data from an ongoing project related to lead exposure in St. Louis City, MO, analyzed the association between aggregate blood lead levels and specific indicators violent crime within the city.

**Design:**

Ecological study.

**Setting:**

St. Louis, Missouri.

**Exposure measure:**

Blood lead levels.

**Main outcome measure:**

Official reports of violent crimes were categorized as 1) crimes involving a firearm (yes/no), 2) assault crimes (with or without a firearm), 3) robbery crimes (with or without a firearm), 4) homicides and 5) rape.

**Results:**

With the exception of rape, aggregate blood-lead levels were statistically significant predictors of violent crime at the census tract level. The risk ratios for each of the outcome measures were as follows: firearm crimes 1.03 (1.03–1.04), assault crimes 1.03 (1.02–1.03), robbery crimes 1.03 (1.02–1.04), homicide 1.03 (1.01, 1.04), and rape 1.01 (0.99–1.03).

**Conclusions:**

Extending prior research in St. Louis, results suggest that aggregated lead exposure at the census tract level predicted crime outcomes, even after accounting for important sociological variables. Moving forward, a more developed understanding of aggregate level crime may necessitate a shift toward studying the synergy between sociological and biological risk factors such as lead exposure.

## Introduction

Lead absorption, both in utero and early across childhood, has been linked to a variety of deleterious outcomes related to overall physiological and neurological growth (for a general review, see) [1]. Regarding neurological outcomes in particular, lead absorption early in development has been prospectively associated with reductions in total brain volume and to reductions in gray matter [2]. These differences, by extension, are thought to impair impulse regulation and to reduce general intelligence for exposed individuals across the remainder of the life course [3–10]. Most recently, Rueben and colleagues [9] sought to further test the association between early life lead exposure and IQ by examining over 500 participants from New Zealand (the Dunedin cohort) tracked across over 30 years of development. After adjusting for a range of confounders (e.g., mother’s intelligence), increased lead exposure was associated with lower overall intelligence in adulthood, as well as lower scores on measures of perceptual reasoning and working memory (but not verbal comprehension and processing speed measures).

The link between lead exposure and neural development becomes particularly important for understanding the development of violent tendencies, as several lines of evidence have found that diminished functioning in the prefrontal cortex is associated with impulsivity as well as with antisocial and violent behavior [11]. Indeed, neurological insults, such as the ones possibly predicted by lead exposure, have been hypothesized to play a key role in the development of serious and persistent criminal conduct [1,8,10]. This relationship was also anticipated by Moffitt’s [12] theoretical work, which suggested early neurological insults meaningfully increase the risk of acting violently across the life course. Seemingly supportive of such a hypothesized link, several studies have reported an association between lead exposure and the risk for a variety of delinquent and antisocial behaviors [13] including criminal conduct at the individual level [4, 8–10, 14]

### Lead exposure & crime in the aggregate

Equally important, however, is the consideration that environmental sources of lead exposure are not uniformly distributed across the population [15]. Rather, lead exposure risk is often concentrated geographically [16–17]. If it is the case that exposure to lead influences behavior at the individual level, then it is plausible that areas with increased levels of ambient lead might jointly experience increased aggregate levels of criminal behavior. One method of testing this possibility is via the use of ambient air-lead levels, and indeed these measures have been linked statistically with a number of aggregate criminal outcomes such as homicide and assault rates [17–18]. Areas with increased levels of lead in the air—across various units of analysis—experienced higher rates of criminal behavior measured in a variety of different ways [17–18]. Yet, it remains premature at this point to reach any strong conclusions about macro-level measures of lead exposure and crime rates due in part to several lingering methodological concerns [16].

Macro-level researchers have long known that spatial autocorrelation, for instance—the tendency for certain outcomes to cluster together geographically for reasons other than a causal association between those two variables—can bias parameter estimates [19]. This potential bias is important because it could lead to incorrect conclusions about the relationship between lead exposure and crime rates. Boutwell et al. [16] drew on macro-level data from St. Louis and uncovered a significant association between blood lead levels and violent, non-violent, and total crime rates across census tracts even after adjusting for potential confounders and spatial autocorrelation. Though suggestive, the effect of aggregate lead levels on broad crime rates obscures the specific types of criminal involvement that lead might impact. Prior macro research examined particularly violent forms of crime (e.g., homicide) [17], yet it remains less clear whether lead exposure raises the risk of only certain types of crime across a wider variety of crime types that it might plausibly impact. In an effort to answer these questions, and to extend existing bodies of evidence, we examined data related to lead exposure measured at the census tract level, and a variety of violent forms of criminal behavior.

## Methods

### Study design

Using data drawn from an ongoing project on urban lead exposure we assessed the association between aggregate blood lead levels and violent crime rates across all 106 Census tracts in St. Louis City Missouri, USA [16, 20]. Concentrated disadvantage, age of the housing stock, and residential mobility were considered important covariates and were included in all statistical models.

### Data

#### Blood lead levels

Children are particularly sensitive to lead burden and appear particularly vulnerable to elevated blood lead levels [21]. Thus, we obtained and geocoded the home addresses of 59,645 children who were less than 72 months in age and who had blood lead level (BLL) tests performed in St. Louis City from 1996 to 2007 by the Missouri DHSS’ Health Strategic Architecture and Information Cooperative (MOHSAIC). The total number of BLL tests were aggregated up to the Census tract level according to the child’s registered address. This resulted in a mean value of 562.3 (SD = 368.4; Min = 17; Max = 1581) BLL tests per census tract. We then calculated the proportion of BLL tests that registered ≥5 mg/dL (for additional detail regarding the risks of lead exposure at similarly low levels and for alternative analytical approaches see) [5, 9–10] within each census tract. The average proportion of high BLL (i.e., ≥5 mg/dL) within each census tract was 0.43 (SD = 0.1; Min = 0.07; Max = 0.74). We should note that the selection of a cutoff was chosen with the recognition that there is no level of exposure that is considered safe for children by the Centers for Disease Control and Prevention. In particular, we relied on prior broad guidelines from the CDC, which has recommended that BLLs ≥5 mg/dL be regarded as high [22] (see also https://www.cdc.gov/nceh/lead/ACCLPP/Final_Document_030712.pdf). Finally, it is important to note that for all analyses presented below, the proportions were transformed into percentages in order to provide more context for the interpretation of effect sizes.

#### Violent crimes

We geocoded the locations of all 15,734 violent crimes (irrespective of the victim’s ages) reported by the St. Louis Metropolitan Police Department (data that ultimately correspond to those submitted to the Federal Bureau of Investigations’ Uniform Crime Report for the years 2010–2012) using ArcGIS version 10.2.2 (ESRI, Redlands, CA) and geographic boundary files from the U.S. Census Bureau (https://www.census.gov/geo/maps-data/data/tiger.html). The Uniform Crime Report defines violent crimes as those that involve force or threat of force, including murder, non-negligent manslaughter, rape, robbery, and aggravated assault (https://www.fbi.gov/about-us/cjis/ucr/crime-in-the-u.s/2013/crime-in-the-u.s.-2013/violent-crime/violent-crime-topic-page/violentcrimemain_final). For the purposes of this research, we further categorized violent crimes as 1) crimes involving a firearm (yes/no), 2) assault (with or without a firearm), 3) robbery (with or without a firearm), 4) homicides and 5) rape. Our decision to investigate these particular crime types was determined by either a paucity of evidence regarding their association with lead (in the case of firearm violence) or a need to replicate prior associations of aggregate lead exposure with violent crimes such as assaults and homicides [17].

We calculated the mean crime rate per census tract for each crime category for the 3-year period from 2010–2012 by dividing the number of crimes by the population size of each census tract. We used the 5-year population estimate from the 2008–2012 American Community Survey (ACS) as the denominator since it is the most reliable population estimate for the 3-year study period [23] (available at: http://www.census.gov/acs/www/. Accessed October 5, 2014.) We also used indirect standardization to calculate the standardized incidence ratio (SIR) for each census tract [15], which represents the observed crime rate relative to the expected crime rate for each crime category.

#### Concentrated disadvantage

We constructed an index of concentrated disadvantage using principal components analysis of eight variables from the 2008–2012 ACS [23]. This measure was constructed combining information about the proportion of the population that was African American, proportion of female-headed households, proportion of households receiving food stamps, proportion of individuals using public health insurance programs (all ages and races), proportion of households with children under age 18, percentage of households without employment during the past 12 months, proportion of households below the federal poverty line, and the median household income (mean-centered). The principal components analysis suggested that one component explained 74.9% of the total variance observed among the 8 variables. The component was standardized and weighted by the estimated loading coefficients (see also) [16, 24].

#### Additional covariates

The mean age of housing units per census tract was drawn from the 2008–2012 ACS in order to account for the persistent source of lead exposure in older homes (primarily through lead-based paints and dust containing lead particles) [25]. In addition, we calculated the proportion of properties that were occupied by renters (also from the ACS) to account for residential mobility between census tracts. Lastly, we categorized all instances of assault and firearm crimes that occurred in domestic settings (1 = yes, 0 = no) from the Uniform Crime Report to be included as an additional covariate at certain stages of the analytical process.

### Statistical analysis

Prior to our multivariate analysis, all covariates were assessed using histograms, Q-Q plots and the Shapiro-Wilk test to assess whether they followed a Gaussian distribution. The population size for each census tract was log-transformed in order to follow the Gaussian distribution and all variables included in the study passed checks of normality. Given the spatial structure of our data, we fit Bayesian sparse spatial generalized linear mixed models (SGLMM) [19] to each of the outcomes described above. The sparse SGLMM introduces a spatial random effect to account for the spatial structure (adjacency) of areal data and greatly improves regression inference relative to the traditional areal mixed model [26] due to how it handles spatial confounding [27]. We defined the spatial adjacency (neighbors) of census tracts using three different methods: rook contiguity (share a common border), queen contiguity (share a common border or point), and using the inverse distance weighted average of the 5 nearest neighboring census tracts.

Model results did not vary substantively by the neighborhood definition so we present results from the less stringent queen contiguity structure here. Specifically, we used the sparse SGLMM to perform spatial Poisson regressions with offset, where the offset for the *i*th census tract was the log-transformed population size of the census tract. This procedure essentially transforms the count-based outcome into a rate. We used the prior distributions recommended by Hughes and Haran [19] and we chose the first *q =* 38 Moran eigenvectors, which virtually exhausted the possible patterns of spatial attraction for the St. Louis City adjacency structure. We drew 100,000 samples from the posterior distribution.

The resulting Monte Carlo standard errors were on the order of 0.001, which indicates convergence of the Markov chain [19]. We used the package ‘ngspatial’ [28–29] in version 3.0.1 of R [29] for all analyses. A large amount of census tracts did not experience a homicide in the time window observed (*n* = 31) and some did not report any rapes (*n* = 13). Thus, we fit a zero-inflated spatial Poisson regression model for these two outcomes using Integrated Nested Laplace Approximation (INLA) methods recommended by Rue et al. [30]. These models yielded similar results to those of the sparse SGLMM, however, we present the results from the zero-inflated models for the homicide and rape outcomes because the INLA models account for over-dispersion and zero counts across census tracts.

All models were adjusted for the aforementioned potential confounders, with the exception that the robbery, homicide, and rape outcomes were not adjusted for domestic settings (as this information was not available). We should note too that we fit spatial negative binomial models to further determine the robustness of the Poisson models, which are presented in the paper. In all instances, the negative binomial models produced a set of results that were very similar to the Poisson models. We then exponentiated the model coefficients (i.e., exp[β]) in order to present relative risks (RR) with their corresponding 95% credible intervals. We also report choropleth maps of the crude crime rates, the crude SIRs, and the adjusted SIRs by quantile (using the predicted values from the multivariate models which adjusted for all confounders and spatial dependence) to show the census tracts with extreme high (low) crime rates.

## Results

**Fig 1:**
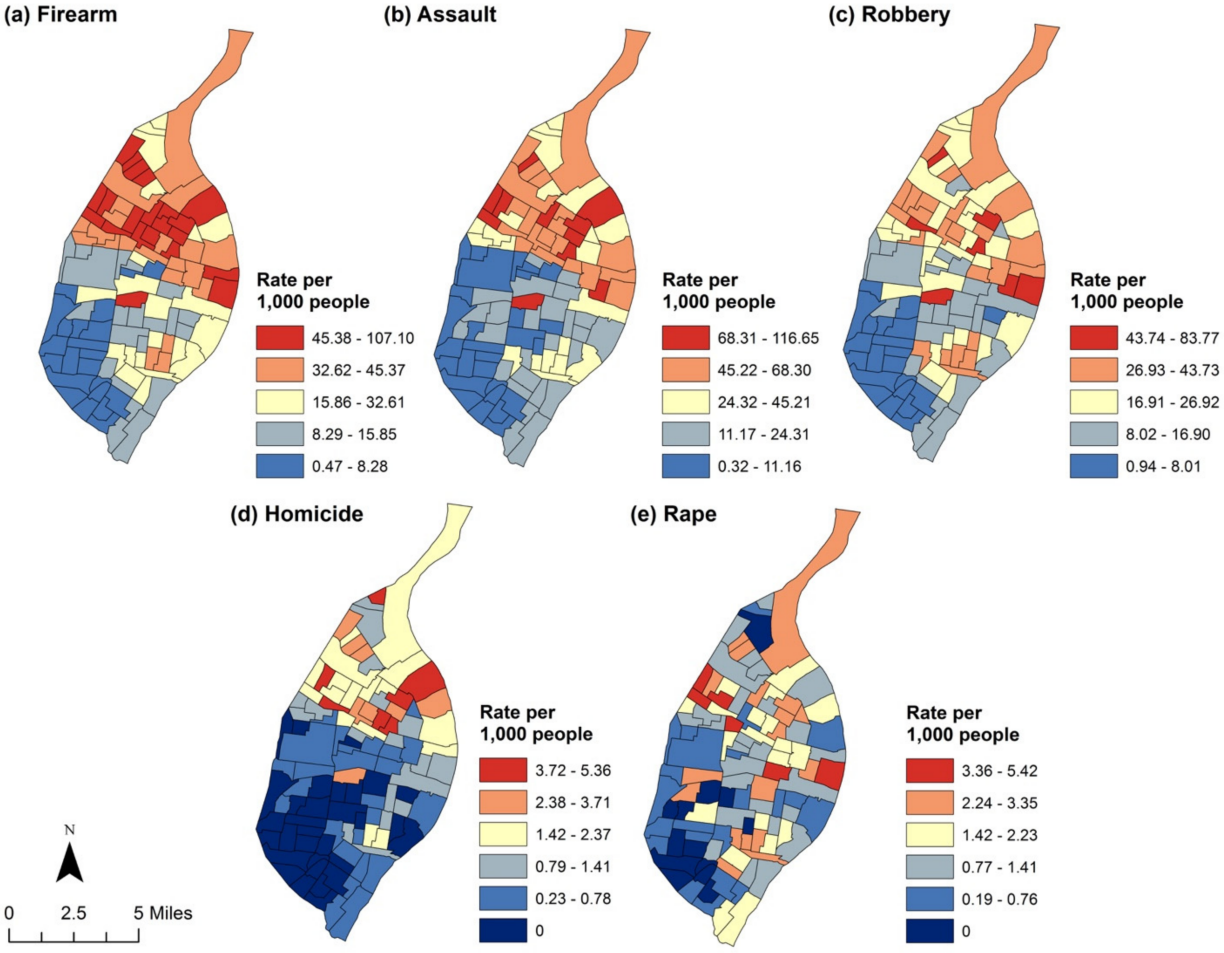
Crude rates of violent crime are presented by quantile, with darker blue shades indicating lower crime rates and darker red shades indicating higher crime rates. Rates are per 1,000 people.

**Fig 2:**
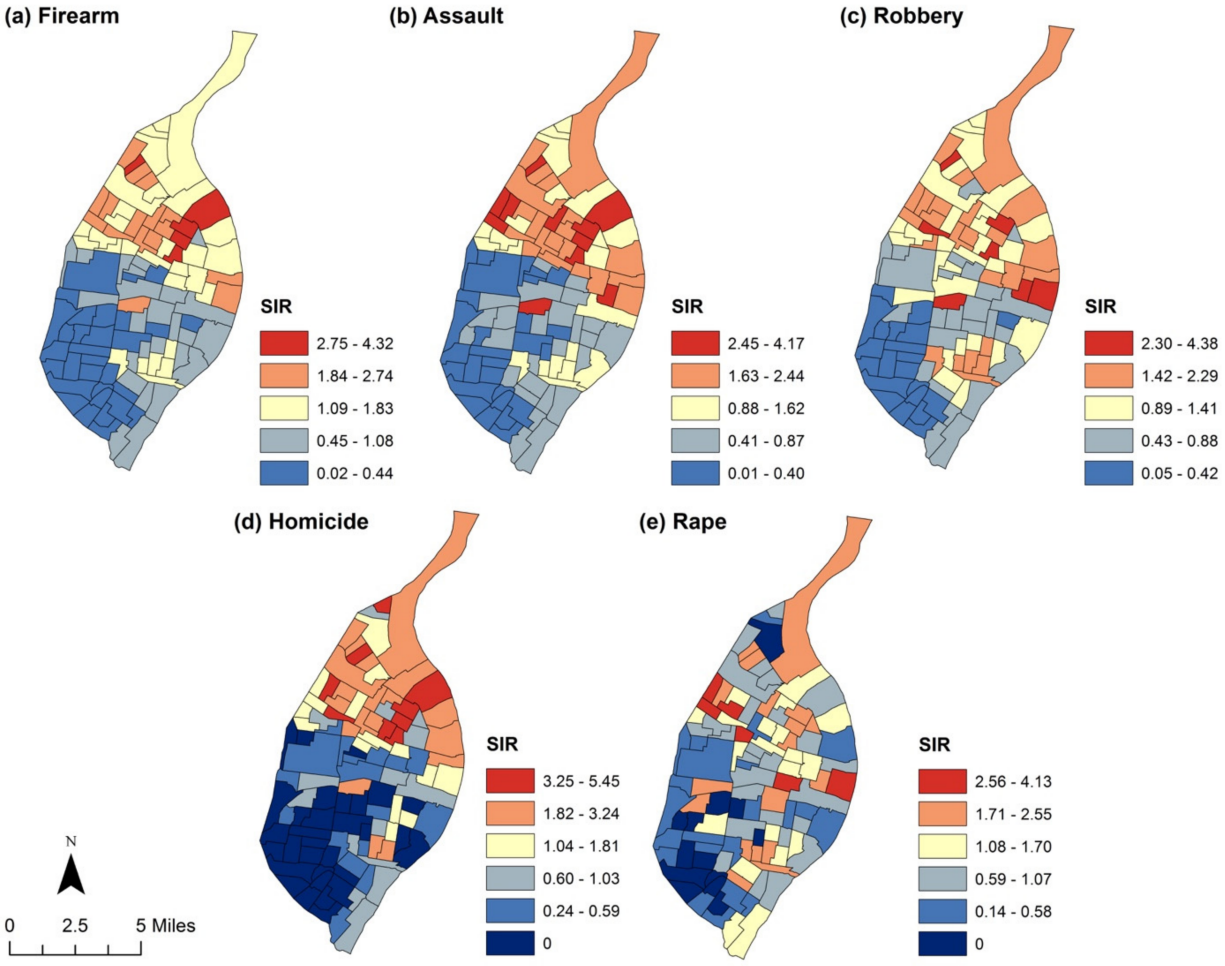
Crude standardized incidence ratios (SIR) are presented by quantile, with darker blue shades indicating lower SIRs and darker red shades indicating higher SIRs.

**Fig 3:**
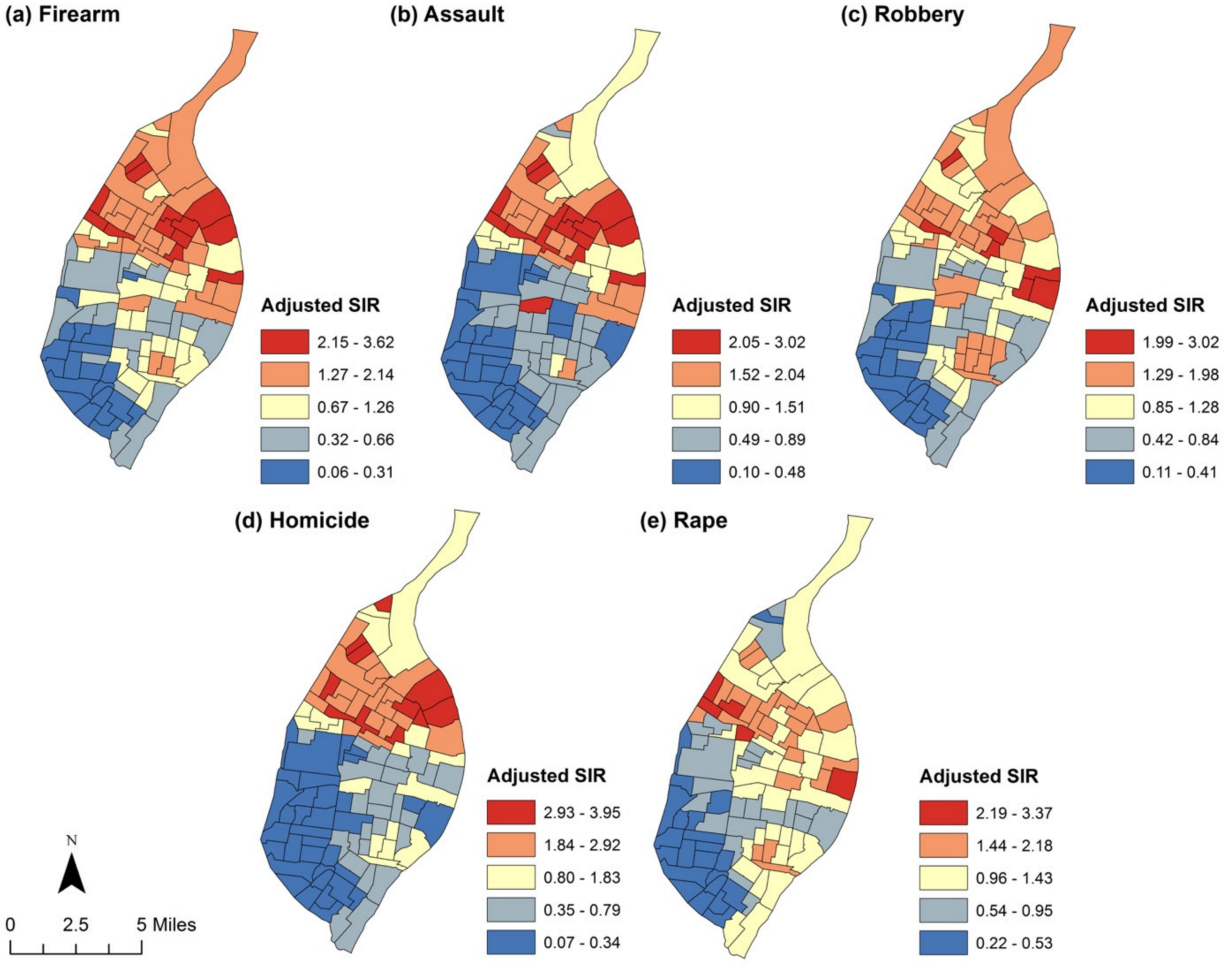
Adjusted standardized incidence ratios (SIRs) presented for each crime type. **Note**: All models include adjustment for the proportion of elevated blood lead level tests, concentrated disadvantage, median housing age, the proportion of renter-occupied housing, domestic settings (except for the robbery and homicide/rape outcomes), and spatial autocorrelation using spatial Poisson regression (firearm, assault, and robbery outcomes) and zero-inflated Poisson regression (homicide and rape outcomes).

Characteristics of the 106 census tracts in St. Louis City are shown in Table 1. Figs 1 and 2 present the geographic distribution of unadjusted crime rates. Generally, crime rates tend to be higher than expected in the northern part of the city, with noticeably lower rates observed for all crime types in the southwestern quadrant of the city. As can be seen in Table 2, census tracts with a higher percentage of children with elevated BLL tests tended to experience higher rates of all crimes analyzed with the exception of rape. Specifically, after controlling for concentrated disadvantage, median housing age, renter-occupied housing and domestic settings, increasing the percentage of elevated BLL tests by just 1 percentage point was associated with a 1.03 (95% CI: 1.03, 1.04) times greater risk for firearm crime, 1.03 (95% CI: 1.02, 1.03) times greater risk for assault crimes, 1.03 (95% CI: 1.02, 1.04) times greater risk for robbery, and 1.03 (95% CI: 1.01, 1.04) times greater risk for homicide (it is perhaps useful to note that the similarity in coefficients is the result of rounding). The relative risk was positive for rape (RR = 1.01, 95% CI: 0.99, 1.03), but was not statistically significant. Fig 3 presents the distribution of the adjusted SIRs by quantile for each crime type across St. Louis City. As can be seen, a stronger contrasting pattern of crimes was revealed after adjustment for confounders. This pattern is particularly apparent among firearm and assault crimes, with census tracts in the northern part of the city having much higher burdens of observed (reported) crimes than expected after adjustment (we also examined the possibility that BLL might interact with concentrated disadvantage to predict crime by including a multiplicative interaction term in our models. This analysis, which is available in supplementary files, did not for the most part reveal a significant interaction effect in the data).

**Table 1:** Characteristics of St. Louis City census tracts (n = 106).

	Mean (SD)
**Mean crime rates per census tract**	
Total violent crime rate (per 1,000)	
Quartile 1 (1.1–23.7)	12.27 (6.30)
Quartile 2 (23.8–46.6)	34.49 (7.25)
Quartile 3 (46.7–79.2)	64.62 (10.67)
Quartile 4 (79.3–174.0)	111.68 (27.8)
Firearm crime rate (per 1,000 people)	
Quartile 1 (0.2–9.3)	4.36 (2.98)
Quartile 2 (9.4–23.0)	15.21 (3.61)
Quartile 3 (23.1–40.5)	32.53 (5.18)
Quartile 4 (40.5–106.0)	57.29 (16.85)
Assault crime rate (per 1,000 people)	
Quartile 1 (0.2–10.9)	6.1 (2.91)
Quartile 2 (11.0–23.4)	16.33 (3.44)
Quartile 3 (23.5–49.3)	36.56 (7.74)
Quartile 4 (49.4–117.0)	69.58 (16.4)
Robbery crime rate (per 1,000 people)	
Quartile 1 (0.9–10.1)	4.99 (3.07)
Quartile 2 (10.2–19.6)	15.08 (2.93)
Quartile 3 (19.7–30.2)	24.01 (2.84)
Quartile 4 (30.3–83.9)	39.68 (12.49)
Homicide rate (per 1,000 people)	
Quartile 1 (0.0–0.0)	0 (0)
Quartile 2 (0.1–0.7)	0.46 (0.15)
Quartile 3 (0.8–1.8)	1.17 (0.34)
Quartile 4 (1.9–5.4)	3.08 (1.16)
Rape rate (per 1,000 people)	
Quartile 1 (0.0–0.5)	0.2 (0.22)
Quartile 2 (0.6–1.1)	0.87 (0.17)
Quartile 3(1.2–2.1)	1.58 (0.29)
Quartile 4 (2.1–5.5)	3.18 (0.93)
**Mean proportion of elevated blood lead tests (≥5 mg/dL)**	
Quartile 1 (0.7–0.31)	0.19 (0.07)
Quartile 2 (0.32–0.45)	0.39 (0.04)
Quartile 3 (0.46–0.57)	0.52 (0.04)
Quartile 4 (0.58–0.74)	0.63 (0.04)
**Mean age of housing units, *years***	67.85 (9.78)
**Mean proportion of renter-occupied housing**	0.53 (0.19)
**Mean population size**	3,005 (1,184)
**Measures of concentrated disadvantage per census tract**	
Proportion of African Americans	0.55 (0.37)
Proportion of female-headed households	0.21 (0.14)
Proportion of households receiving food stamps	0.28 (0.17)
Proportion of public health insurance users	0.37 (0.15)
Proportion of households with children <18 years of age	0.22 (0.09)
Percentage of households without employment past 12 months	0.16 (0.09)
Proportion of households below the federal poverty line	0.27 (0.13)
Median household income	$33,841 ($13,418)

**Table 2:** Associations between lead exposure and violent crime types, St. Louis City, MO.

	Firearm crimes*		Assault crimes		Robbery crimes		Homicide*		Rape*	
Characteristic	RR	95% CI	RR	95% CI	RR	95% CI	RR	95% CI	RR	95% CI
Proportion of elevated blood lead tests	1.03	(1.03, 1.04)	1.03	(1.02, 1.03)	1.03	(1.02, 1.04)	1.03	(1.01, 1.04)	1.01	(0.99, 1.03)
Concentrated disadvantage	1.13	(1.06, 1.20)	1.17	(1.12, 1.24)	1.06	(1.01, 1.11)	1.71	(1.29, 2.27)	1.09	(0.86, 1.38)
Median housing age	1.00	(0.99, 1.01)	1.01	(1.00, 1.01)	1.01	(1.00, 1.01)	1.01	(0.99, 1.03)	1.01	(0.99, 1.03)
Proportion of renter-occupied housing	3.41	(2.76, 4.22)	2.45	(2.00, 2.95)	4.57	(3.59, 5.74)	1.62	(0.53, 5.05)	3.94	(1.41, 10.71)
Domestic Setting	1.02	(1.02, 1.03)	1.02	(1.02, 1.03)	—	—	—	—	—	—

*Estimates were derived from spatial Poisson regression models for firearm, assault and robbery crimes. Estimates for homicide and rape were derived from zero-inflated Poisson models. CI indicates a credible interval.

## Discussion

We expanded on prior work that has examined the association between blood lead levels and crime rates in the city of St. Louis, Missouri [16]. In particular, we categorized total violent crimes into firearm-related offenses, assaults, robbery, homicide, and rape in order to test whether the association between particular types of crime and aggregate measures of BLL differs across crime type. After adjusting for potential confounders and simultaneously accounting for spatial autocorrelation, the results provided evidence of an association between the proportion of elevated blood-lead exposure and various incarnations of violence across census tracts in the city (we should reiterate that “elevated” refers to the cut-point designated for the current analysis, based on prior guidance from the CDC). In general, census tracts with a higher proportion of BLL’s above 5 mg/dL experienced more gun crimes, assaults, robberies, and homicides than those with lower lead levels.

The results, however, should be interpreted cautiously. As Boutwell and colleagues [16] have noted, aggregate level studies on the association between lead exposure and violence are limited and methodologically complex. Though beyond the scope of the current study, future designs capable of examining both individual *and* macro-level data [31] will be helpful in further testing the association between lead exposure and violence. Also worth considering are the limitations inherent in officially recorded crime data (i.e., official police reports). The obvious issue is that these measures include only crimes known to the police and thus may miss many instances of criminal behavior [32]. As a result, it remains unknown whether our findings would replicate using self-reported data. To date, there has been no effort (of which we are aware) to examine the effects of aggregate level lead exposure on aggregate level self-reported crime. An additional point worth considering is that it is impossible to discern whether the individuals who reported high levels of lead exposure were also the individuals responsible for the crimes captured in the data. The individual measures, in this case, were meant to serve as proxies for ambient lead exposure across census tracts. In other words, to the extent that a given census tract is contaminated with ambient lead (via aging housing stock, etc.), that should be reflected to some extent in the people residing within that space.

A final important limitation concerns the nature of the measure used to assess macro level lead exposure. Recall that the measure was comprised of individual-level blood tests that were then aggregated to the census tract level. An obvious difficulty with a measure of this nature is that the individuals tested for lead exposure were not randomly drawn from the population, thus not everyone in the city possessed an equal likelihood of being tested and included in the analysis. The primary consequence stemming from this sort of selection is that the results of our analysis may not represent the effect of aggregate lead exposure on crime in a manner that can be widely generalized. Stated another way, to the extent that our sample differs from the general population, our findings may reflect those differences. As a result, caution should be used when interpreting the results observed herein.

In an attempt to further probe potential sources of bias in our paper, we examined whether the number of lead tests administered in a census tract was related to the proportion of respondents with elevated BLLs. This is relevant because more tests in a given area might reflect greater lead levels, or a greater tendency among those residents to seek testing. Thus, we might expect that a greater number of lead tests in a given census tract could predict a higher proportion of respondents with elevated BLLs. Additional sensitivity analyses intended to explore this issue, however, did not appear to suggest that the number of samples drawn in a given census tract was associated with an elevated (for the purposes of the current study) BLL test result.

With these caveats in mind, the current study represents advances over prior research in that it specifically examines various types of criminal violence, including gun violence, which is consistently of interest to researchers across fields [33]. To the best of our knowledge, this is one of the first studies to directly examine the association between a measure of macro-level blood lead and an indicator of firearm crimes in addition to homicide and rape [17–18, 34]. Ultimately, our results dovetail in important ways with prior work, and further underscore the potential association between lead and criminality regardless of level of analysis.

## Supporting information

S1 Table Associations between lead exposure and violent crime types with interaction terms, St. Louis City, MO. (DOCX)

S1 Fig Crude rates of violent crime are presented by quantile, with darker shades indicating higher crime rates and lighter shades indicating lower crime rates. Rates are per 1,000 people.
(DOCX)

S2 Fig Crude standardized incidence ratios (SIR) are presented by quantile, with darker shades indicating higher SIRs and lighter shades indicating lower SIRs. (DOCX)

S3 Fig Adjusted standardized incidence ratios (SIRs) presented for each crime type. (DOCX)
