# Supplementary material for: Aggregate-level lead exposure, gun violence, homicide, and rape
Source: PLoS One. 2017 Nov 27;12(11):e0187953. doi: 10.1371/journal.pone.0187953 (PMC5703470; doi:10.1371/journal.pone.0187953)
Supplement: S1 Table — (DOCX) [file pone.0187953.s001.docx]

|  | | | | | | | | | | |
| --- | --- | --- | --- | --- | --- | --- | --- | --- | --- | --- |
|  |  |  |  |  |  |  |  |  |  |  |
|  | **Firearm crimes*** | | **Assault crimes** | | **Robbery crimes** | | **Homicide*** | | **Rape*** | |
| **Characteristic** | **RR** | **95% CI** | **RR** | **95% CI** | **RR** | **95% CI** | **RR** | **95% CI** | **RR** | **95% CI** |
| Proportion of elevated blood lead tests | 1.03 | (1.03, 1.04) | 1.03 | (1.02, 1.03) | 1.03 | (1.02, 1.03) | 1.03 | (1.01, 1.05) | 1.01 | (0.99, 1.03) |
| Concentrated disadvantage | 1.07 | (0.94, 1.22) | 0.94 | (0.84, 1.05) | 0.98 | (0.87, 1.12) | 1.66 | (0.85, 3.24) | 1.07 | (0.61, 1.88) |
| Median housing age | 1.00 | (0.99, 1.01) | 1.00 | (0.99, 1.01) | 1.00 | (0.99, 1.01) | 1.01 | (0.99, 1.03) | 1.01 | (0.99, 1.03) |
| Proportion of renter-occupied housing | 3.37 | (2.76, 4.18) | 2.37 | (1.97, 2.84) | 4.08 | (3.27, 5.11) | 1.64 | (0.54, 5.10) | 3.93 | (1.39, 10.77) |
| Domestic Setting | 1.02 | (1.02, 1.03) | 1.02 | (1.02, 1.03) | --- | --- | --- | --- | --- | --- |
| BLL * CD Interaction | 1.00 | (0.99, 1.01) | 1.01 | (1.01, 1.02) | 1.00 | (0.99, 1.01) | 1.01 | (0.99, 1.01) | 1.00 | (0.99, 1.01) |

**S1. Associations between lead exposure and violent crime types with interaction terms, St. Louis City, MO**
