# Supplementary material for: Aggregate-level lead exposure, gun violence, homicide, and rape
Source: PLoS One. 2017 Nov 27;12(11):e0187953. doi: 10.1371/journal.pone.0187953 (PMC5703470; doi:10.1371/journal.pone.0187953)

**S1 Fig.** Crude rates of violent crime are presented by quantile, with darker shades indicating higher crime rates and lighter shades indicating lower crime rates. Rates are per 1,000 people.


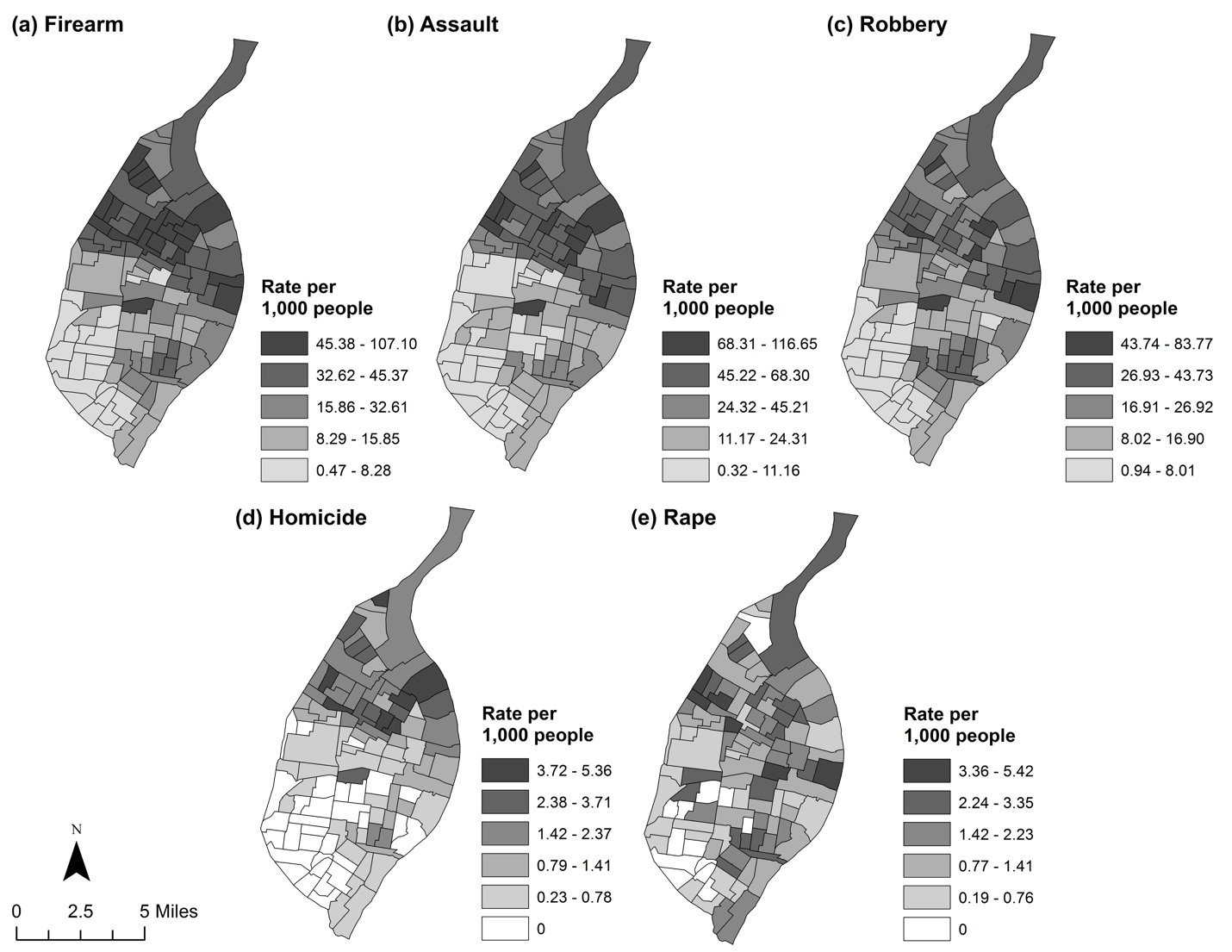

Supplement: S1 Fig — Rates are per 1,000 people. (DOCX) [file pone.0187953.s002.docx]
