# Supplementary material for: Aggregate-level lead exposure, gun violence, homicide, and rape
Source: PLoS One. 2017 Nov 27;12(11):e0187953. doi: 10.1371/journal.pone.0187953 (PMC5703470; doi:10.1371/journal.pone.0187953)

**S2 Fig.** Crude standardized incidence ratios (SIR) are presented by quantile, with darker shades indicating higher SIRs and lighter shades indicating lower SIRs.


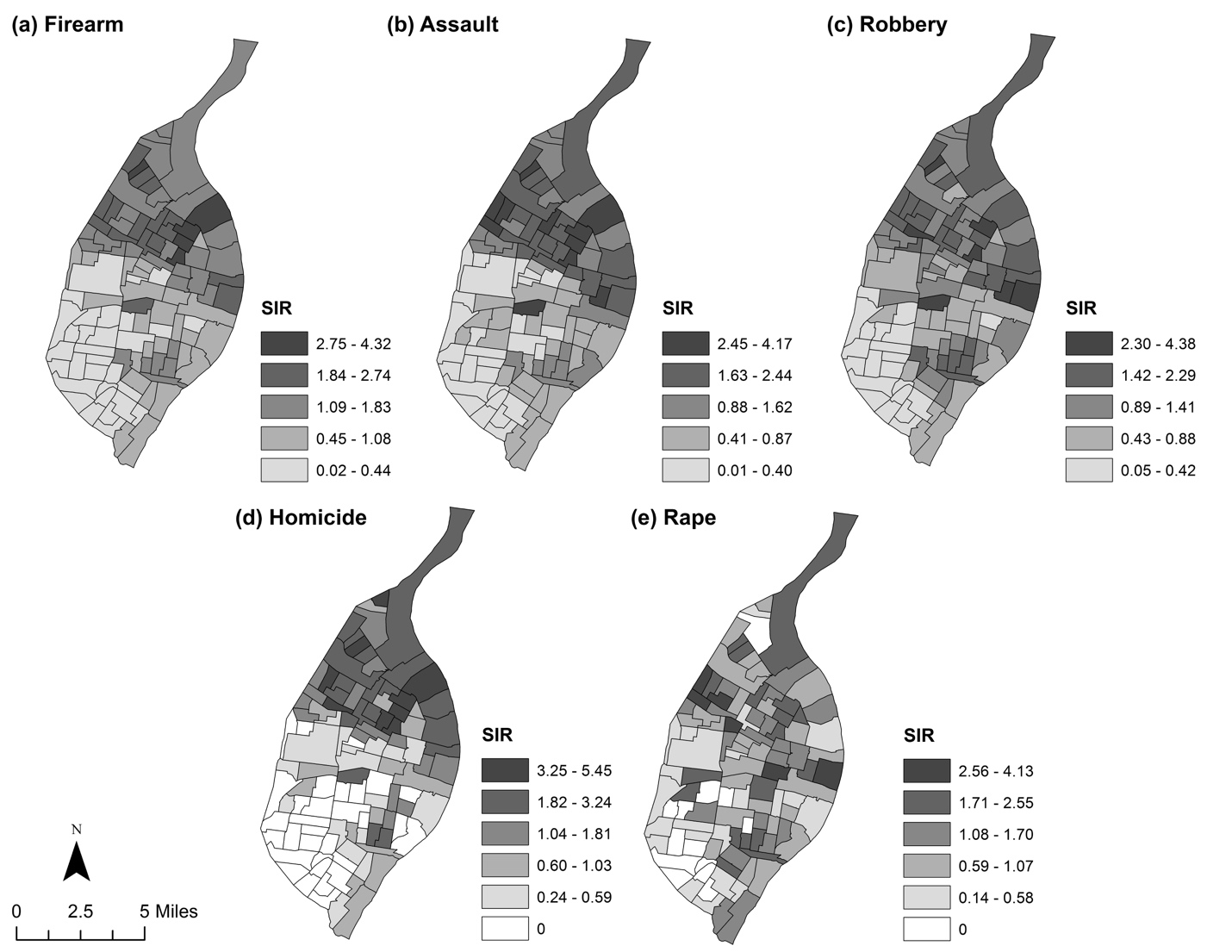

Supplement: S2 Fig — (DOCX) [file pone.0187953.s003.docx]
