# Supplementary material for: Aggregate-level lead exposure, gun violence, homicide, and rape
Source: PLoS One. 2017 Nov 27;12(11):e0187953. doi: 10.1371/journal.pone.0187953 (PMC5703470; doi:10.1371/journal.pone.0187953)
Supplement: S3 Fig — (DOCX) [file pone.0187953.s004.docx]

**S3 Fig.** Adjusted standardized incidence ratios (SIRs) presented for each crime type.


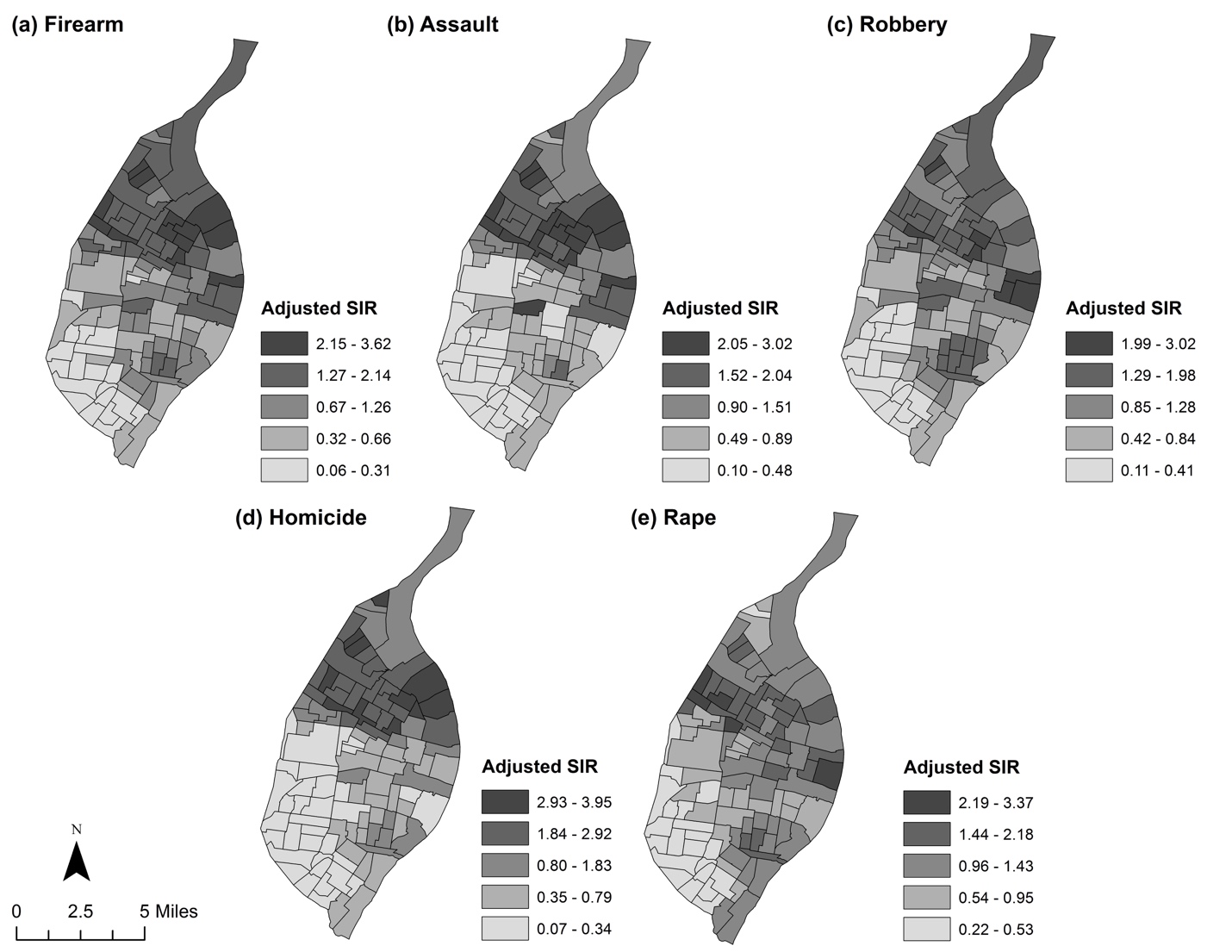


**Note:** All models include adjustment for the proportion of elevated blood lead level tests, concentrated disadvantage, median housing age, the proportion of renter-occupied housing, domestic settings (except for the robbery and homicide/rape outcomes), and spatial autocorrelation using spatial Poisson regression (firearm, assault, and robbery outcomes) and zero-inflated Poisson regression (homicide and rape outcomes).
